# Identification of racial disparities across MammaPrint and BluePrint subtypes in HR + HER2- breast cancer

**DOI:** 10.1038/s41523-026-00932-1

**Published:** 2026-03-19

**Authors:** Sonya Reid, Lindsay Venton, Jennifer G. Whisenant, Anne E. Weidner, Jennifer Wei, Harshini Ramaswamy, Christa Dreezen, Nicole Chmielewski-Stivers, Andrea R. Menicucci, William Audeh, Tuya Pal

**Affiliations:** 1 Vanderbilt University Medical Center, Nashville, TN USA; 2 Agendia Inc, Irvine, CA USA; 3 Agendia NV, Amsterdam, Netherlands

**Keywords:** Cancer, Oncology, Risk factors

## Abstract

We compared clinicopathologic features, MammaPrint and BluePrint molecular subtype, and outcomes by race among females with hormone receptor-positive (HR+), HER2- early-stage breast cancer (EBC). Of 1018 participants with HR+ HER2- EBC enrolled from two registries (BEST and FLEX), 509 White females were propensity score matched 1:1 to 509 Black females based on age and/or menopausal status. MammaPrint classified tumors as High-Risk or Low-Risk; and together with BluePrint, classified tumors as Luminal A-Type, Luminal B-Type, or Basal-Type. Recurrence-free survival (RFS) was analyzed by race and molecular subtype. Cox proportional hazards models assessed association of clinicopathologic features with outcomes. Basal-Type tumors were more prevalent among Black vs White participants (11.0% vs 4.8%, *p* < 0.001). Independent of race, participants with Basal-Type tumors had lower 3-year RFS (83.7%) compared to Luminal B-Type (93.7%) and Luminal A-Type (96.5%, *p* < 0.0001). Multivariate analysis revealed that participants with High-Risk, Luminal B- and Basal-Type tumors had significantly worse 3-year outcomes compared to Low-Risk Luminal A-Type, after controlling for race and potential confounders. Genomic classification identified higher proportions of High-Risk HR+ HER2- EBC among Black participants. Molecular subtype was independently prognostic of 3-year survival, supporting the prognostic and potentially predictive importance of genomic testing to reduce racial survival disparities among Black females with EBC.

## Introduction

Hormone receptor-positive (HR+), human epidermal growth factor receptor 2-negative (HER2-) breast cancer is the most common subtype across all racial and ethnic groups. In the United States, Black females have a 5% lower incidence of breast cancer compared to White females, yet have 40% higher mortality rates^1^. The largest proportion of the Black-White breast cancer survival disparity is due to lower survival among Black women with HR + HER2- breast cancer, with more pronounced differences in those under 50 years of age^1,2^. Contributors to racial survival disparities include social determinants of health, patterns of care, and biological factors (e.g., immunohistochemistry [IHC] subtype, molecular subtype, tumor and germline genomics)^2–4^. Recognizing that lower socioeconomic status is not synonymous with race, lower socioeconomic status and systemic racism contribute to higher mortality rates^1–5^, and Black females continue to have higher breast cancer mortality rates compared to their White counterparts, even after controlling for socioeconomic factors^6^. Taken together, while socioeconomic status-related factors contribute to worse survival outcomes, they do not fully account for survival disparities observed in Black females.

Furthermore, Black females with HR + HER2- breast cancer have worse clinical outcomes despite receiving comparable systemic therapy, suggesting that biological factors may partly contribute to the racial survival disparity observed in HR+ HER2- breast cancer^4^. However, it should be noted that biological differences may also be a result of lifestyle factors, nutritional, environmental exposures and genetic influences^6^. Few studies have evaluated the role of tumor genomic differences in survival across diverse populations, largely due to the underrepresentation of non-European ancestry populations in clinical trials and population-based studies. Genomic assays, which comprehensively capture tumor biology, are widely used in early-stage breast cancer to provide prognostic and sometimes predictive information beyond standard IHC-classifications^7–9^. However, inequities in the access or utilization of genomic assays may further exacerbate racial disparities in breast cancer outcomes if not adequately addressed^10,11^. Understanding the biological and tumor genomic differences by race could improve treatment decisions and promote optimal care for Black females with early-stage breast cancer, ultimately improving long-term outcomes. In this study, we utilized RNA-based expression analyses through the MammaPrint risk of recurrence signature and BluePrint molecular subtyping signature to examine whether tumor genomic differences contribute to racial survival disparities among women with HR + HER2- breast cancer.

## Results

### Clinical characteristics and genomic risk summary

Clinical characteristics for participants with HR + HER2- tumors are summarized in Table 1. The median age was 54 years, and most participants had T1-T2, node negative, grade 2–3 tumors. Black participants were significantly more likely than White participants to have nodal involvement (29.9% vs. 17.5%; *p* < 0.001), higher grade tumors (76.5% vs. 69.9%; *p* < 0.001), and receive chemotherapy (73.6% vs. 47.1%; *p* < 0.001).

**Table 1:** Clinical characteristics for participants with HR + HER2- tumors

	Black	White	Overall	*P*-value
	(*N* = 509)	(*N* = 509)	(*N* = 1018)	
**Age (Years)**				
Mean (SD)	54 (±13)	54 (±13)	54 (±13)	0.983
**Menopausal Status**				
Post-	250 (51.9%)	251 (52.1%)	501 (52.0%)	1
Pre-/Peri-	232 (48.1%)	231 (47.9%)	463 (48.0%)	
**Tumor Size**				
T1	203 (56.7%)	202 (63.9%)	405 (60.1%)	0.645
T2	124 (34.6%)	94 (29.7%)	218 (32.3%)	
T3	24 (6.7%)	17 (5.4%)	41 (6.1%)	
T4	7 (2.0%)	3 (0.9%)	10 (1.5%)	
**Lymph Node Status**				
N0	241 (70.1%)	240 (82.5%)	481 (75.7%)	< 0.001
N1	77 (22.4%)	47 (16.2%)	124 (19.5%)	
N2	20 (5.8%)	1 (0.3%)	21 (3.3%)	
N3	6 (1.7%)	3 (1.0%)	9 (1.4%)	
**Grade**				
G1	113 (23.5%)	143 (30.0%)	256 (26.8%)	< 0.001
G2	225 (46.8%)	259 (54.4%)	484 (50.6%)	
G3	143 (29.7%)	74 (15.5%)	217 (22.7%)	
**Treatment Type**				
Neoadjuvant therapy	77 (20.5%)	73 (14.6%)	150 (17.1%)	0.251
Adjuvant therapy	290 (77.1%)	416 (83.2%)	706 (80.6%)	
Non-surgical	9 (2.4%)	11 (2.2%)	20 (2.3%)	
**Chemotherapy**				
Yes	259 (73.6%)	160 (47.1%)	419 (60.5%)	< 0.001
No	93 (26.4%)	180 (52.9%)	273 (39.5%)	
**ER Staining (%)**				
Weak Positive (1–10%)	20 (4.1%)	11 (2.2%)	31 (3.1%)	0.257
Positive (>10%)	469 (95.9%)	492 (97.8%)	961 (96.9%)	

Data represented as *N* (%), unless otherwise specified. Participants were matched by age and menopausal status. Differences in groups were assessed by using Pearson’s Chi-squared tests or Fisher’s exact test (for categorical variables) or Student’s t-test (for numerical variables). Statistical significance was defined as *p* < 0.05. Unknown values excluded. *HR* *+* , hormone receptor-positive, *HER2-* human epidermal growth factor receptor 2-negative, *N* number of participants, *ER* estrogen receptor.

**Fig. 1:**
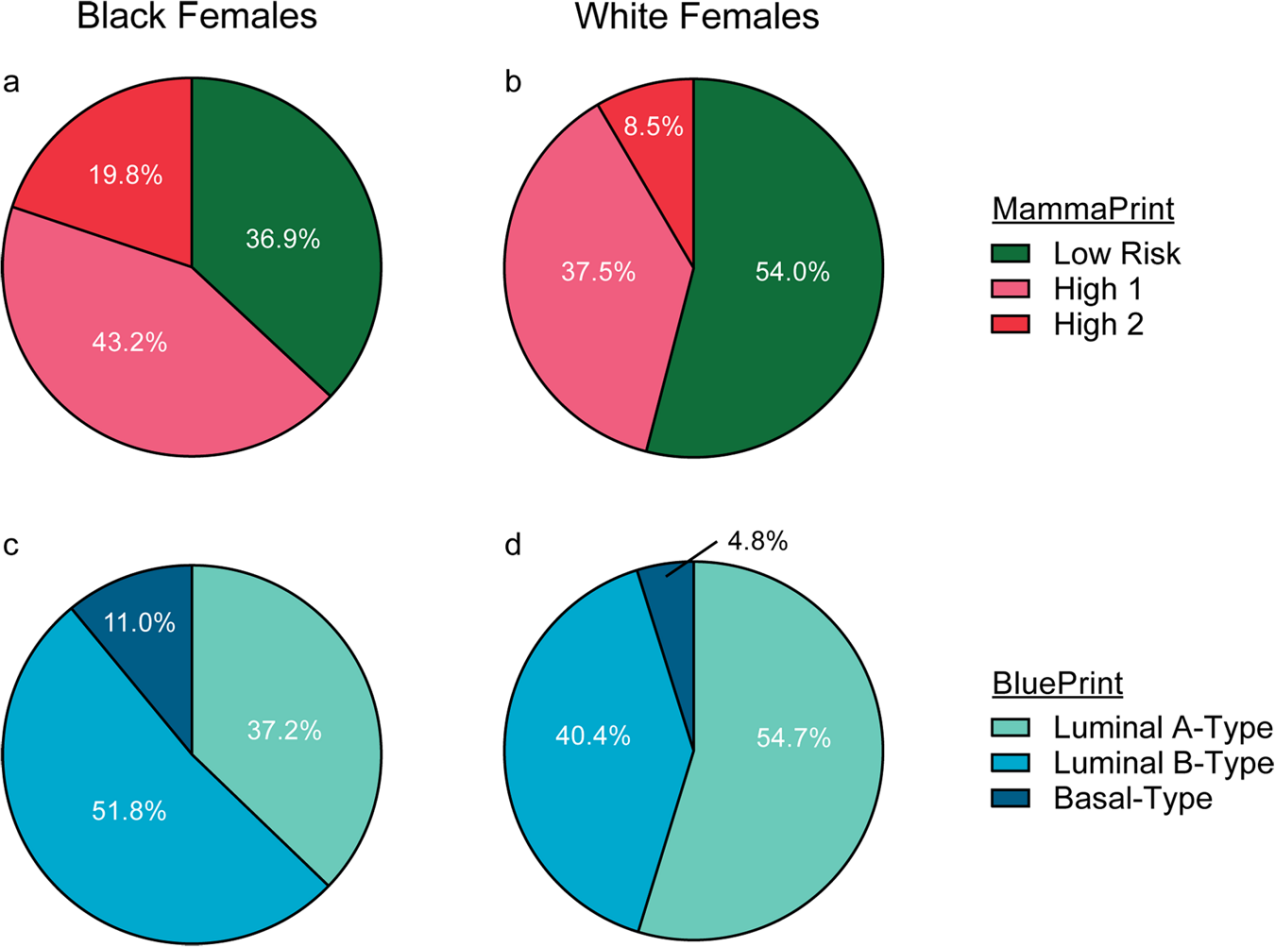
MammaPrint and BluePrint percent distribution by race in participants with HR + HER2- tumors. MammaPrint risk results in (**a**) Black (*N* = 509) and (**b**) White (*N* = 509) females, stratified by Low Risk (green), High Risk 1 (pink) and High Risk 2 (red). BluePrint subtype distribution in (**c**) Black (*N* = 492) and (**d**) White (*N* = 475) females, stratified by Luminal A-Type (light green), Luminal B-Type (light blue) and Basal-Type (dark blue). Significance between groups was assessed by Chi-squared test. Statistical significance was defined as *p* < 0.05. BluePrint results were available for *N* = 492 Black and *N* = 475 White participants. Participants with genomically HER2-Type tumors were removed from analyses due to the small number of participants with available data (*N* = 5). *HR* + , hormone receptor-positive, *HER2-* human epidermal growth factor receptor 2-negative, *N* number of participants.

Fewer Black females had genomic Low-Risk, Luminal A-Type (37.2%) tumors compared with White females (54.7%; *p* < 0.001; Fig. 1a–d, Supplemental Fig. 1). In contrast, H2 tumors were significantly more prevalent among Black females (19.8%, *N* = 101/509) compared to White females (8.4%, *N* = 43/509; *p* < 0.001). Additionally, Black females had a higher proportion of Luminal B-Type tumors compared to White females (51.8%, *N* = 255/492 versus 40.4%, *N* = 192/475; *p* = 0.002), and more than twice as many Black females had genomically Basal-Type tumors compared with White females (11.0%, *N* = 54/492 versus 4.8%, *N* = 23/475; *p* < 0.001).

Among Basal-Type tumors, 45.0% were ER weak positive (1-10%), while 55.0% were ER positive (>10%), for both Black and White participants (Supplemental Table 1). Black females had more grade 3 Basal-Type tumors (100%), compared to 77.3% for White females, however, not all grade 3 tumors were Basal-Type.

When stratified by ER IHC staining category (1–10%, 11–50%, 51–90%, and 91–100%), distinct distributions of BluePrint molecular subtypes were observed across ER expression levels in both Black and White participants (Supplemental Fig. 2). Basal-Type tumors were most prevalent among participants with ER weak positive tumors (1–10%), however, were still observed within 11–50% and 51–90% ER% staining in both racial groups.

### Association of 3-year RFS with MammaPrint and BluePrint

**Fig. 2:**
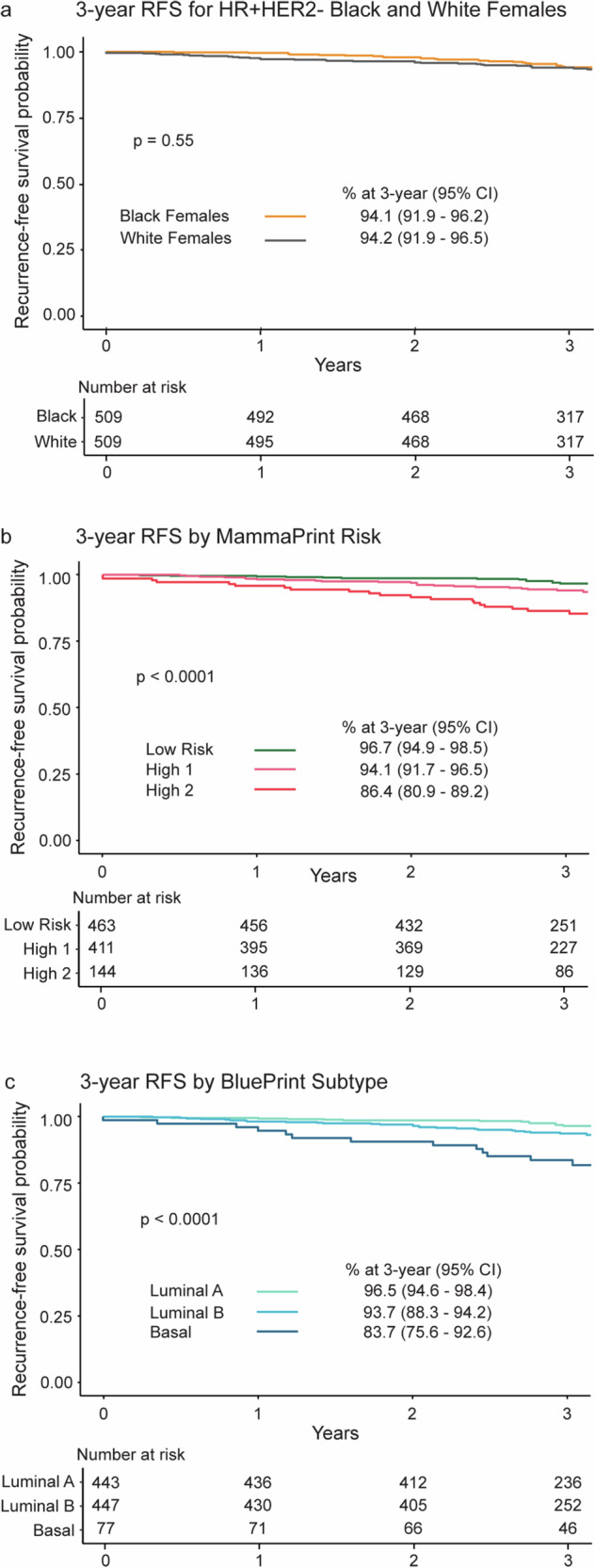
Association of Race, MammaPrint, and BluePrint with 3-year RFS in participants with HR + HER2- tumors. 3-year RFS was stratified by (**a**) race, (**b**) MammaPrint Low (green), High 1 (pink), and High 2 (red) Risk groups, and (**c**) combined MammaPrint and BluePrint subtypes, Luminal A-Type (light green), Luminal B-Type (light blue) and Basal-Type (dark blue). Participants with genomically HER2-Type tumors were removed from all follow up analyses due to the small number of participants with available data (*N* = 5). Significance between groups was assessed by log-rank test. Statistical significance was defined as *p* < 0.05. RFS recurrence-free survival; HR + hormone receptor-positive, HER2- human epidermal growth factor receptor 2-negative, CI confidence interval.

The unstratified 3-year RFS rate was 94.1% (95% CI 91.9–96.2) for Black females and 94.2% (95% CI 91.9–96.5) for White females (*p* = 0.55; Fig. 2a). When stratified by MammaPrint, the 3-year RFS was 96.7% (95% CI 94.9–98.5) for Low-Risk tumors, 94.1% (95% CI 91.7–96.5) for H1, and 86.4% (95% CI 80.9–89.2) for H2 tumors (*p* < 0.0001; Fig. 2b). BluePrint classification revealed the highest 3-year RFS in females with Luminal A-Type tumors at 96.5% (95% CI 94.6–98.4), followed by those with Luminal B-Type tumors at 93.7% (95% CI 88.3–94.2). The worst outcomes were observed for females with Basal-Type tumors, with an RFS of 83.7% (95% CI 75.6–92.6; *p* < 0.0001; Fig. 2c).

**Fig. 3:**
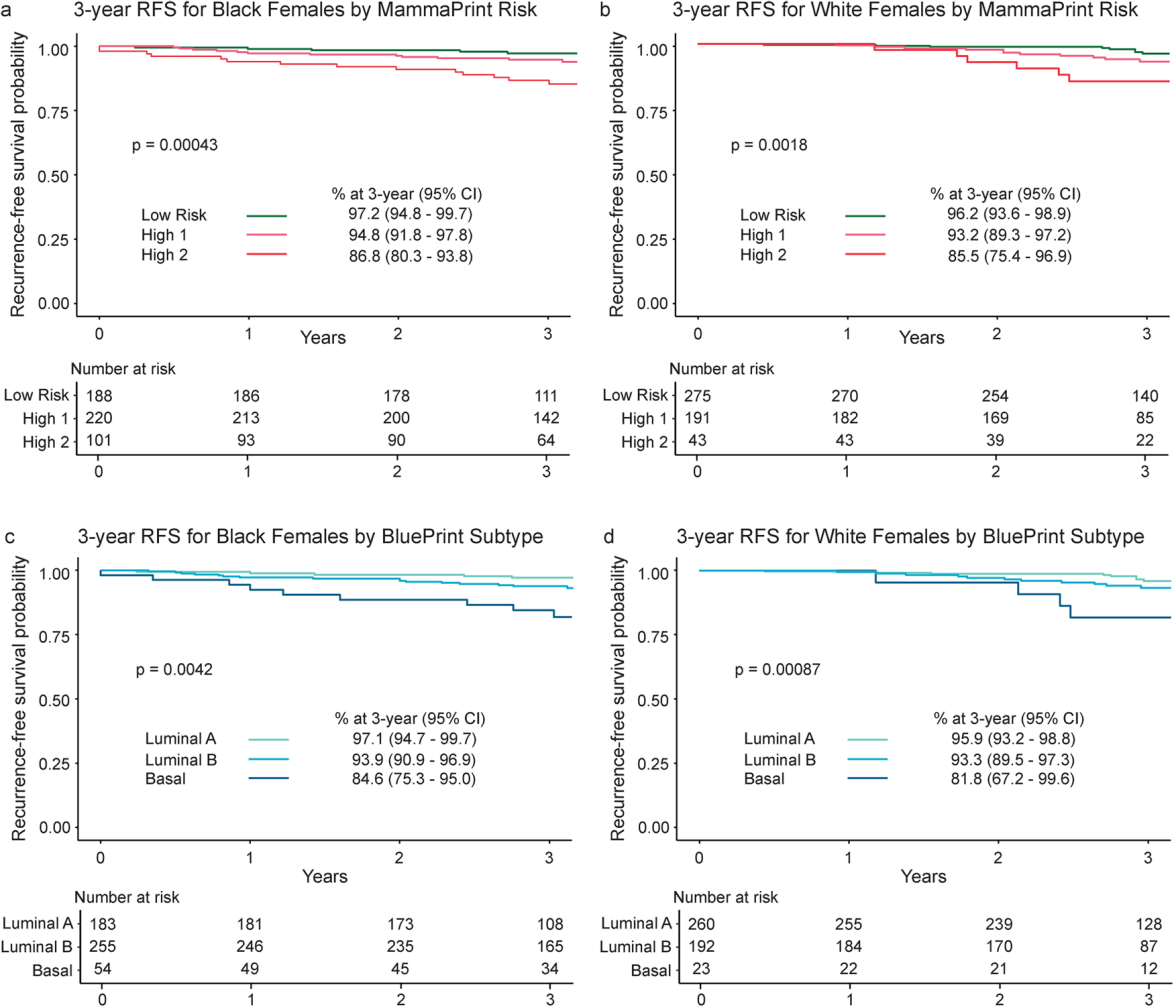
Association of MammaPrint and BluePrint with 3-year RFS by race in females with HR + HER2- tumors. 3-year RFS in (**a**) Black and (**b**) White participants were stratified by MammaPrint into Low (green), High 1 (pink), and High 2 (red) Risk groups. 3-year RFS in (**c**) Black and (**d**) White participants were then stratified by molecular subtype with MammaPrint and BluePrint. MammaPrint and BluePrint categorize tumors as Luminal A-Type (light green), Luminal B-Type (light blue) and Basal-Type (dark blue). Participants with genomically HER2-Type tumors were removed from all follow up analyses due to the small number of participants with available data (*N* = 5). Significance between groups was assessed by log-rank test. RFS recurrence-free survival, HR + hormone receptor-positive, HER2- human epidermal growth factor receptor 2-negative, CI confidence interval.

Comparable 3-year RFS trends were observed across MammaPrint and BluePrint stratification for each race (Fig. 3). Among Black females, 3-year RFS differed significantly by MammaPrint risk group, with the highest RFS observed in participants with Low-Risk tumors (97.2%; 95% CI 94.8–99.7), followed by those with High 1 (94.8%; 95% CI 91.8–97.8), and the lowest RFS among High 2 (86.8%; 95% CI 80.3–93.8; *p* = 0.00043; Fig. 3a). White females had 3-year RFS of 96.2% (95% CI 93.6–98.9) for Low-Risk tumors, 93.2% (95% CI 89.3–97.2) for High 1, and 85.5% (95% CI 75.4–96.9) for High 2 tumors (*p* = 0.0018; Fig. 3b).

For BluePrint, Black females with Luminal A-Type tumors experienced the most favorable outcomes, with a 3-year RFS of 97.1% (95% CI 94.7–99.7), followed by those with Luminal B-Type tumors at 93.9% (95% CI 90.9–96.9). Participants with Basal-Type tumors had the lowest 3-year RFS at 84.6% (95% CI 75.3–95.0; p = 0.0042; Fig. 3c). Consistent findings were observed among White females, with 3-year RFS rates of 95.9% (95% CI 93.2–98.8) for Luminal A-Type tumors, 93.3% (95% CI 89.5–97.3) for Luminal B-Type tumors, and 81.8% (95% CI 67.2–99.6) for Basal-Type tumors (*p* = 0.00087; Fig. 3d).

### 3-year RFS Multivariate Analysis by MammaPrint and BluePrint, Race and Clinical Variables

In a multivariate model adjusting for MammaPrint/BluePrint, age, menopausal status, race, tumor size, nodal stage, grade, and ER % staining, unsurprisingly the presence of 2-3 lymph nodes (LN 2/3) was significantly associated with worse 3-year RFS (HR: 5.46, 95% CI: 2.16–13.81, *p* < 0.001) and pre-/peri-menopausal status trended towards worse outcomes (HR: 3.56, 95% CI: 1.17–10.88, *p* = 0.056). Notably, participants with High-Risk Luminal B- and Basal-Type tumors were over 5 times (HR: 5.08, 95% CI: 1.66–9.54, *p* = 0.004) and over 10 times (HR: 10.82, 95% CI: 2.18–23.67, *p* = 0.004) more likely, respectively, to have a recurrence within 3 years compared to participants with Luminal A-Type tumors, while race had no association with outcome (Table 2). Within this population, when receipt of chemotherapy was included in the model, only LN 2/3 had a significant association with 3-year RFS. MammaPrint/BluePrint was likely no longer significant due to the collinearity of receipt of chemotherapy and genomic risk (Supplemental Table 2). Similarly, in the subset of participants who received chemotherapy, only LN 2/3 remained significantly associated with 3-year RFS. The lack of an observed association between MammaPrint/BluePrint and outcomes in the chemotherapy-treated subset likely reflects limited statistical power due to the small sample size (Supplemental Table 3).

**Table 2:** Univariate and multivariate Cox proportional hazards regression analysis of factors associated with recurrence-free survival

Variable	HR (univariate)	HR (multivariate)
**MammaPrint/BluePrint Group**		
Low Risk/Luminal A–		
High Risk/Luminal B	2.22 (1.24–4.00, *p* = 0.008)	5.08 (1.66–9.54, *p* = 0.004)
High Risk/Basal	4.84 (2.38–9.82, *p* < 0.001)	10.82 (2.18–23.67, *p* = 0.004)
**Age**		
Mean (SD)	1.00 (0.98–1.02, *p* = 0.768)	1.09 (0.95–1.14, *p* = 0.742)
**Menopausal Status**		
Post–		
Pre-/Peri-	1.00 (0.51–1.58, *p* = 0.985)	3.56 (1.17–10.88, *p* = 0.056)
**Race**		
White-		
Black	1.17 (0.70–1.93, *p* = 0.551)	1.15 (0.36–1.64, *p* = 0.494)
**Tumor Size**		
T1-		
T2/3	2.70 (1.51–4.83, *p* = 0.001)	1.98 (0.95–4.12, *p* = 0.067)
**Lymph Node Status**		
N0-		
N1	2.65 (1.44–4.87, *p* = 0.002)	1.86 (0.84–4.11, *p* = 0.124)
N2/3	5.22 (2.42–11.29, *p* < 0.001)	5.46 (2.16–13.81, *p* < 0.001)
**Grade**		
G1-		
G2	3.95 (1.39–11.19, *p* = 0.010)	1.16 (0.37–3.65, *p* = 0.802)
G3	7.72 (2.71–21.99, *p* < 0.001)	1.23 (0.35–4.32, *p* = 0.748)
**ER% Staining**		
Weak Positive (1–10%)-		
Positive (>10%)	0.38 (0.16–0.88, *p* = 0.024)	1.16 (0.30–4.42, *p* = 0.828)

Data represented as HRs (95% CI, *p*-value). *p* < 0.05 indicates significant risk factor. T4 tumors not included due to small sample size (*N* = 10). *HR* hazard ratio, *CI* confidence interval, *RFS* recurrence-free survival.

### Association of MammaPrint and BluePrint with 10-year RFS and OS in Black females (BEST study only)

**Fig. 4:**
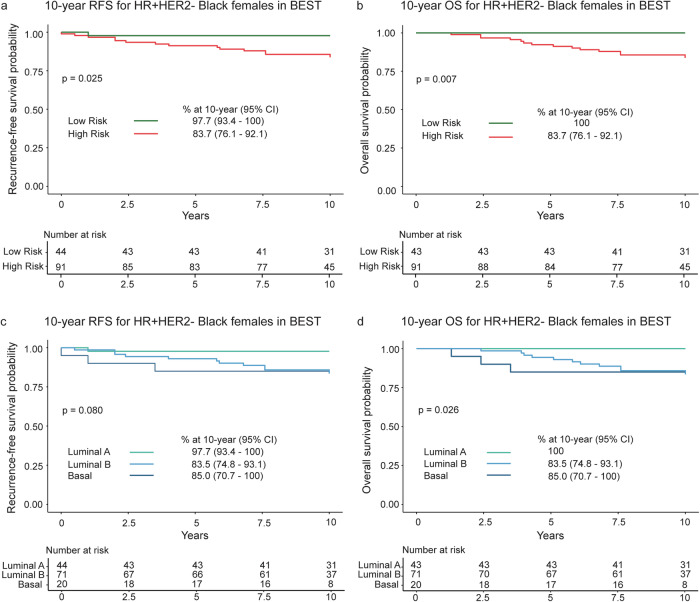
Association of MammaPrint and BluePrint with 10-year RFS and OS in Black females with HR + HER2- tumors. 10-year (**a**) RFS and (**b**) OS were stratified by MammaPrint into Low Risk (green) and High Risk (red) of distant metastasis for Black participants in BEST. 10-year (**c**) RFS and (**d**) OS were then stratified by molecular subtype with MammaPrint and BluePrint for all Black participants in BEST. MammaPrint and BluePrint categorize tumors as Luminal A (light green), Luminal B (light blue), or Basal (dark blue). One participant was excluded from OS endpoint due to missing event data. Participants with genomically HER2-Type tumors were removed from all follow up analyses due to the small number of participants with available data (*N* = 5). Significance between groups was assessed by log-rank test. Statistical significance was defined as *p* < 0.05. RFS recurrence-free survival, OS overall survival, HR + hormone receptor-positive, HER2- human epidermal growth factor receptor 2-negative, CI confidence interval.

The unstratified 10-year RFS and OS rates were 88.4% (95% CI 82.9–94.2) and 89.0% (95% CI 83.7–94.7), respectively. When further classified by MammaPrint, participants with Low-Risk tumors had significantly higher 10-year RFS (*N* = 44; 97.7%, 95% CI 93.4–100) and OS (*N* = 43; 100%) compared to those with High-Risk tumors, who had a 10-year RFS of 83.7% (*N* = 91; 95% CI 76.1–92.1, *p* = 0.025) and OS of 83.7% (*N* = 91; 95% CI 76.1–92.1, *p* = 0.007) (Fig. 4a, b).

By BluePrint classification, the 10-year RFS was highest in participants with Luminal A-Type tumors at 97.7% (95% CI 93.4–100), followed by 83.5% (95% CI 74.8–93.1) in Luminal B-Type tumors and 85.0% in Basal-Type tumors (95% CI 70.7–100) (*p* = 0.080; Fig. 4c). Participants with Luminal A-Type tumors also had significantly higher 10-year OS (100%) compared to participants with Luminal B (83.5%; 95% CI 74.8–93.1) and Basal-Type tumors (85.0%; 95% CI 70.7–100) (*p* = 0.026; Fig. 4d).

## Discussion

In this study of HR + HER2- breast cancer, our findings show over-representation of Basal and Luminal B subtypes based on MammaPrint and BluePrint molecular subtyping among Black compared to White females. Moreover, survival outcomes differed by genomic subtype with lowest survival among Basal and Luminal B subtypes, independent of race.

Consistent with prior research^5,6,12–16^, we observed a higher proportion of genomically High-Risk tumors (MammaPrint H2; BluePrint Luminal B and Basal-Type) among Black compared to White females. Notably, more than twice as many Black females had tumors classified as genomically Basal-Type compared with White females. HR+ Basal subtypes exhibit similar genomic profiles to that of triple-negative breast cancer (TNBC)^17^. Recent whole-transcriptome analyses of Basal-Type tumors from I-SPY 2 participants have shown a stronger concordance of gene expression profiles between HR+ Basal-Type and TNBC, compared to Basal- and Luminal-Type cancers^18,19^, suggesting shared fundamental biology. Importantly, patients with HR+ Basal-type tumors had higher rates of recurrences within 3 years, similar to those with TNBC^20–22^. Furthermore, studies suggest HR+ Basal-Type tumors not only resemble TNBC biologically^19,23^ but may also exhibit resistance to endocrine therapy^24^. These findings highlight the critical need in identifying patients with HR+ Basal-Type tumors, as these patients have similar rates of metastasis, as well as chemo- and immunotherapy sensitivity, to TNBC^7,25–28^.

In contrast to prior studies which have shown an association between HR+ Basal-Type tumors and weak (1–10%) ER staining^29^, we found more than half (55%) of Basal-Type tumors had greater than 10% ER staining, in both Black and White participants. These results question the functional activity of the estrogen receptor among patients with HR+ breast cancer. Previous analyses of ER+ tumors from NBRST participants revealed a significantly higher occurrence of the ERΔ7 dominant-negative splice variant in Basal-Type compared to Luminal-type tumors^27^. The ERΔ7 splice variant, associated with ER dysfunction and endocrine therapy resistance, is indistinguishable from functioning ER through IHC. Consequently, our findings underscore the importance of incorporating genomic assays to identify and manage HR+ tumors with transcriptional activity characteristic of HR- tumors. Given the higher incidence of Basal-Type tumors among Black participants observed in this study, it is possible that Black females may have a greater frequency of tumors harboring the ERΔ7 splice variant relative to White females, though this hypothesis needs further investigation.

In the current study, stratification of breast cancer subtypes by MammaPrint and BluePrint revealed distinct outcomes in a propensity score matched cohort with 3-year follow-up. Both MammaPrint and BluePrint subtyping had equivalent prognostic performance across race. Specifically, females with H2 and Basal-Type tumors had significantly worse outcomes compared to Luminal A-Type tumors, regardless of race. Long-term survival data further highlighted disparities among Black females. At 10 years, those with High-Risk Basal- and Luminal B-Type tumors continued to show significantly worse outcomes compared to those with Low-Risk Luminal A-Type tumors.

Distinct outcomes after stratification of early-stage HR + HER2- breast cancer by MammaPrint and BluePrint observed here are consistent with the 5-year survival outcomes reported in the NBRST study^20,22^. Furthermore, these findings are similar to studies of predominantly White cohorts, such as the prospective MINDACT trial, which reported an excellent 8-year OS probability of 95.7% for patients with clinically high-risk, but genomically Low-Risk tumors who did not receive chemotherapy^30,31^. Additionally, a recent analysis of the National Cancer Database (NCDB) of females with HR + HER2- early-stage breast cancer, including 530 Non-Hispanic Black and 5,040 Non-Hispanic White participants, reported a 95.6% OS rate among Black participants with MammaPrint Low-Risk tumors and no significant survival differences between race when stratified into MammaPrint Low- or High-Risk categories^16^.

These findings emphasize the critical role of genomic subtyping in understanding and addressing disparities in breast cancer outcomes across racial groups. While MammaPrint and BluePrint demonstrated consistent prognostic accuracy between Black and White females in this study, previous analyses using other genomic assays, including the 21-gene Recurrence Score (RS), have revealed differences in tumor biology and survival outcomes across racial groups^32–35^. A large NCDB study comparing 115,651 Non-Hispanic White and 10,814 Non-Hispanic Black participants with ER+ breast cancer using 21-gene Recurrence Score (RS) data highlighted that Black participants were more likely to be diagnosed with high-risk recurrence score tumors (RS ≥ 26) with the worst outcomes. However, Black females with low and intermediate-risk tumors (RS < 26) had significantly worse OS compared to other racial/ethnic groups, despite correcting for recurrence score category^15^. Similarly, an analysis of the SEER database reported that among patients with tumors classified as RS < 26, Black patients had worse survival outcomes compared with White patients, suggesting that the RS threshold may need to be recalibrated for Black patients^36^.

While genomic assays have traditionally been used to identify high-risk tumors to provide prognostic and sometimes predictive information, recent studies indicate their potential to inform future treatment strategies^37^. A recent analysis of participants from the FLEX study with HR + HER2- MammaPrint High Risk breast cancer showed that patients with MammaPrint H2 tumors derived greater benefit from anthracycline-based chemotherapy regimens compared to those with H1 tumors^37^. Furthermore, emerging research has shown improved pathological Complete Response (pCR) rates with chemo-immunotherapy in early high-risk HR + HER2- breast cancer, particularly in tumors with low to moderate ER expression^38–40^.

Despite advancements in treatment options to improve breast cancer outcomes, socioeconomic factors, such as later stage at diagnosis and lack of access to genomic testing, continue to contribute to racial survival disparities^3,6,10^. However, socioeconomic factors alone are unlikely to fully account for survival disparities observed among Black females. These data support the hypothesis that tumor biology in part may play a significant role resulting in racial survival disparities^6,13,15,41–43^. Although identifying the underlying mechanisms leading to higher incidences of high-risk disease in Black females is beyond the scope of this study, these data suggest that molecular subtyping could help elucidate and address the biological differences contributing to these disparities.

The current study has several strengths, including being amongst the largest cohorts of Black participants with HR + HER2- early-stage breast cancer with available gene expression, clinical and survival data. Despite these strengths, there remain some limitations, including the observational study design across two separate cohorts. Consequently, treatment was administered at the discretion of physicians’ standard-of-care guidelines at the time of diagnosis. Furthermore, the period of diagnosis for the cohorts differed, thus we could not match based on year of diagnosis. However, endocrine therapy and/or chemotherapy remained standard of care between 2005 to 2021, thus we do not believe our results are confounded given there were no significant changes in breast cancer management in this timeframe. Additionally, we acknowledge that 3-year outcomes primarily captures early recurrence events and does not reflect late recurrences typical of HR+ breast cancer. The 10-year outcomes observed in the BEST cohort should be interpreted with caution, as this analysis was limited to a smaller, younger cohort of Black females. This study lacked data on treatment adherence, social determinants of health, and detailed comorbidity information, all of which may contribute to observed outcome differences and could not be accounted for in the analyses. This analysis was limited to participants who self-identified as Black or White; outcomes in other racial or ethnic groups could not be evaluated and warrant further investigation.

Molecular subtype classification in this study highlights racial differences in the distribution of genomically High-Risk HR + HER2- early breast cancer. Utilizing genomic assays, such as MammaPrint and BluePrint, are important to identify patients with high-risk Luminal B and Basal-Type tumors, which are over-represented among Black females. Moreover, these subtypes are associated with poorer outcomes compared to Luminal A-Type tumors and may require more aggressive treatment strategies. Furthermore, our findings demonstrate that survival outcomes at 3-years were driven by molecular subtype, independent of race, after controlling for potential confounders. These data suggest tumor genomic testing for all patients may help guide treatment decisions to ultimately reduce racial survival disparities among Black females with breast cancer.

## Methods

### Participant cohort

This analysis included 1,018 female participants with stage I–III, HR + HER2– breast cancer enrolled in one of two observational studies: the Black Women with Breast Cancer: Etiology, Survival and Treatment Outcomes (BEST) study^12,44^ or the Full-genome Data Linked with Clinical Data to Evaluate New Gene Expression Profiles (FLEX) study (ClinicalTrials.gov, NCT03053193, study registration: 2017-02-15).

The BEST study is a population-based observational cohort designed to investigate breast cancer etiology, treatment, and outcomes among Black females. BEST participants were identified through state cancer registries in Tennessee and Florida diagnosed between 2005 and 2015. Eligibility criteria required a diagnosis of invasive breast cancer at age ≤ 50 years. For the present analysis, 139 Black females from the BEST study who met eligibility criteria and had available tumor gene expression data were included.

The FLEX study is an ongoing multi-center, prospective observational study designed to understand how genomic profiling (i.e. MammaPrint, BluePrint, and whole-transcriptome analysis) informs prognosis, guides treatment decisions, and impacts clinical outcomes. Participants with non-metastatic invasive breast cancer who receive MammaPrint with or without BluePrint as standard-of-care are eligible to be enrolled in FLEX. From FLEX, 370 Black females diagnosed between 2017 and 2021 who met eligibility criteria were included.

A comparison cohort of White females was identified from FLEX using 1:1 propensity score matching to Black participants from FLEX and BEST based on age at diagnosis using the MatchIt R package (version 4.4.1)^45^. When age was unavailable, matching was performed based on menopausal status at diagnosis.

Across both studies, race was self-reported and categorized as Black or White. Participants were excluded if they self-identified as mixed race, were male or of unspecified gender, had missing tumor receptor status, multifocal or bilateral disease, or had less than three years of follow-up. Both studies followed ethical principles outlined in the Declaration of Helsinki^46^ and adhere to international Good Clinical Practice (GCP) guidelines^47^. The BEST study was approved by the Institutional Review Board at Vanderbilt University Medical Center (protocol #170233), and the FLEX study was approved by the WCG Institutional Review Board (protocol #2017003). All participants provided written informed consent.

### Clinical and molecular classification

HR and HER2 tumor receptor status were locally assessed using IHC or IHC/fluorescence in situ hybridization, respectively, following established guidelines^48,49^. Participants enrolled in FLEX received standard-of-care MammaPrint testing, with or without BluePrint, to guide treatment decisions^50,51^. For BEST participants, MammaPrint and BluePrint testing were conducted retrospectively after enrollment using previously extracted tumor RNA and therefore, these results did not guide treatment decisions.

The MammaPrint 70-gene signature classified tumors as Low-Risk (MammaPrint index > 0) or High-Risk (MammaPrint index ≤ 0) for distant recurrence^50,52^. High-Risk tumors were further stratified into High Risk 1 (H1: MammaPrint index ≤ 0 to −0.570) or High Risk 2 (H2: MammaPrint index ≤ −0.570 to −1)^53,54^. The BluePrint 80-gene signature classified tumors as Luminal-Type, HER2-Type, or Basal-Type^51^. BluePrint Luminal-Type tumors were further differentiated by MammaPrint to Luminal A-Type (Low-Risk) or Luminal B-Type (High-Risk)^51^.

### Study endpoints

The primary endpoint was 3-year recurrence-free survival (RFS), defined as the time from diagnosis to date of recurrence (local-regional or distant), death from any cause, or censored at the last follow-up date, in accordance with STEEP criteria^55^. Among Black participants in the BEST study with available 10-year follow-up, we also evaluated 10-year RFS and overall survival (OS) by MammaPrint and BluePrint. OS was defined as the time from diagnosis to death from any cause or censored at the last follow-up, per STEEP criteria^55^. Participants with BluePrint HER2-Type tumors were excluded from follow-up analyses due to small numbers (*N* = 5).

### Statistical analysis

Descriptive statistics were used to summarize clinical characteristics and genomic results, using Pearson’s Chi-squared or Fisher’s exact tests for categorical variables, and Student’s t-test for continuous variables. Survival analyses were conducted with a median follow-up of 3-years, with survival curves estimated using the Kaplan-Meier method, with differences assessed by the log-rank test^56^. A Cox proportional hazards model analyzed the association of MammaPrint/BluePrint, age (continuous), menopausal status, race, T size (T1 and T2/3), nodal status (N0, N1, and N2/3), grade, and ER staining (weak positive 1–10% and positive > 10%) with survival outcomes. Variable selection for the Cox regression model was guided by both statistical association with RFS in univariate analyses and clinical relevance based on established prognostic evidence. Proportional hazards assumptions were assessed using Schoenfeld residuals. Results were presented as hazard ratios with 95% confidence intervals and p-values. Statistical significance was defined as a two-sided *p*-value of *p* < 0.05 for all tests. All statistical analyses were conducted using R (version 4.4.1) and SAS (version 9.4; SAS Institute Inc, Cary, NC).

## Supplementary information

BEST_FLEX_Supplementary only_revision_26Feb26

## Data Availability

The datasets generated and/or analyzed during the current study are not publicly available due to contractual and informed consent restrictions but are available from the corresponding author on reasonable request, subject to institutional review and a data use agreement.

## Code availability

The statistical analysis code for this study is not publicly available but may be made available to qualified researchers on reasonable request from the corresponding author.
