# Supplementary material for: Identification of racial disparities across MammaPrint and BluePrint subtypes in HR + HER2- breast cancer
Source: NPJ Breast Cancer. 2026 Mar 19;12:68. doi: 10.1038/s41523-026-00932-1 (PMC13153423; doi:10.1038/s41523-026-00932-1)
Supplement: Supplementary file 1 — BEST_FLEX_Supplementary only_revision_26Feb26 [file 41523_2026_932_MOESM1_ESM.pdf]

Supplementary Information

TITLE: Identification of racial disparities across MammaPrint and BluePrint subtypes in HR+HER2- breast cancer

	BluePrint Subtype										
	Black Participants					White Participants					
**ER Staining (%)**	**Luminal A (N=183)**	**Luminal B (N=255)**	**Basal (N=54)**	**HER2 (N=4)**	**Not Requested (N=13)**	**Luminal A (N=260)**	**Luminal B (N=192)**	**Basal (N=23)**	**HER2 (N=1)**	**Not Requested (N=33)**	**P-value**
Weak Positive (1-10%)	1 (0.6%)	1 (0.4%)	18 (45.0%)	0 (0%)	0 (0%)	1 (0.4%)	0 (0%)	9 (45.0%)	0 (0%)	1 (3.0%)	<0.001
Positive (>10%)	180 (99.4%)	250 (99.6%)	22 (55.0%)	4 (100%)	13 (100%)	258 (99.6%)	190 (100%)	11 (55.0%)	1 (100%)	32 (97.0%)	
**Grade**											
G1	75 (44.1%)	36 (14.9%)	0 (0%)	0 (0%)	2 (15.4%)	113 (47.1%)	23 (12.7%)	0 (0%)	0 (0%)	7 (21.2%)	<0.001
G2	84 (49.4%)	131 (54.1%)	0 (0%)	2 (50.0%)	8 (61.5%)	122 (50.8%)	111 (61.3%)	5 (22.7%)	1 (100%)	21 (63.6%)	
G3	11 (6.5%)	75 (31.0%)	52 (100%)	2 (50.0%)	3 (23.1%)	5 (2.1%)	47 (26.0%)	17 (77.3%)	0 (0%)	5 (15.2%)	

Supplemental Table 1. Sensitivity analysis for ER% staining and Grade, stratified by race and BluePrint subtype. Data represented as N (%). Patients were matched by age and menopausal status. Differences in groups were assessed by Pearson's Chi-squared tests or Fisher's exact test. Forty-six participants from FLEX did not receive BluePrint testing (Not Requested). Statistical significance was defined as p<0.05. Unknown values excluded. Abbreviations: N, number of participants; ER, estrogen receptor.

Variable	HR (univariate)	HR (multivariate)
**MammaPrint/BluePrint Group**		
Low Risk/Luminal A	-	-
High Risk/Luminal B	2.22 (1.24-4.00, p=0.008)	3.67 (0.97-11.92, p=0.056)
High Risk/Basal	4.84 (2.38-9.82, p<0.001)	3.10 (0.26-8.73, p=0.371)
**Age**		
Mean (SD)	1.00 (0.98-1.02, p=0.768)	1.08 (0.94-1.14, p=0.530)
**Menopausal Status**		
Post-	-	-
Pre-/Peri-	1.00 (0.51-1.58, p=0.985)	4.02 (0.97-6.63, p=0.055)
**Race**		
White	-	-
Black	1.17 (0.70-1.93, p=0.551)	0.79 (0.18-1.23, p=0.125)
**Tumor Stage**		
T1	-	-
T2/3	2.70 (1.51-4.83, p=0.001)	2.25 (0.95-5.34, p=0.065)
**Lymph Node Status**		
N0	-	-
N1	2.65 (1.44-4.87, p=0.002)	1.89 (0.74-4.81, p=0.184)
N2/3	5.22 (2.42-11.29, p<0.001)	6.40 (1.98-15.66, p=0.002)
**Grade**		
G1	-	-
G2	3.95 (1.39-11.19, p=0.010)	1.31 (0.28-6.09, p=0.730)
G3	7.72 (2.71-21.99, p<0.001)	1.44 (0.28-7.43, p=0.665)
**ER % Staining**	-	-
Weak Positive (1-10%)		
Positive (>10%)	0.38 (0.16-0.88, p=0.024)	0.97 (0.09-11.10, p=0.982)
**Chemotherapy**		
Yes	-	-
No	2.26 (1.09-4.72, p=0.029)	2.12 (0.43-10.52, p=0.357)

Supplemental Table 2. Univariate and multivariate Cox proportional hazards regression analysis of factors affecting recurrence-free survival including chemotherapy regimen. Patients were matched by age and menopausal status. Data represented as HR (95% CI, p-value). p<0.05 indicates significant risk factor. Abbreviations: HR, hazard ratio; CI, confidence interval; RFS, recurrence-free survival.

Variable	HR (univariate)	HR (multivariate)
**MammaPrint/BluePrint Group**		
Low Risk/Luminal A	-	-
High Risk/Luminal B	1.42 (0.54-3.72, p=0.478)	3.66 (0.80-16.73, p=0.094)
High Risk/Basal	1.87 (0.59-5.89, p=0.286)	3.09 (0.23-21.51, p=0.394)
**Age**		
Mean (SD)	0.99 (0.96-1.02, p=0.393)	1.05 (0.92-1.12, p=0.176)
**Menopausal Status**		
Post-	-	-
Pre-/Peri-	1.00 (0.57-2.01, p=0.985)	5.54 (1.31-8.34, p=0.052)
**Race**		
White	-	-
Black	0.64 (0.32-1.30, p=0.219)	0.75 (0.20-1.62, p=0.295)
**Tumor Stage**		
T1	-	-
T2/3	2.32 (1.06-5.10, p=0.036)	2.03 (0.81-5.08, p=0.129)
**Lymph Node Status**		
N0	-	-
N1	2.21 (1.00-4.85, p=0.049)	2.03 (0.75-5.46, p=0.163)
N2/3	4.20 (1.64-10.79, p=0.003)	6.19 (1.88-15.34, p=0.003)
**Grade**		
G1	-	-
G2	4.31 (0.58-32.32, p=0.155)	1.97 (0.25-15.63, p=0.519)
G3	5.48 (0.73-41.12, p=0.098)	2.07 (0.25-17.38, p=0.501)
**ER % Staining**		
Weak Positive (1-10%)	-	-
Positive (>10%)	0.84 (0.20-3.52, p=0.814)	1.09 (0.08-11.54, p=0.992)

Supplemental Table 3. Univariate and multivariate Cox proportional hazards regression analysis of factors affecting recurrence-free survival within a chemo treated population. Patients were matched by age and menopausal status. Data represented as HR (95% CI, p-value). p<0.05 indicates significant risk factor. Abbreviations: HR, hazard ratio; CI, confidence interval; RFS, recurrence-free survival.

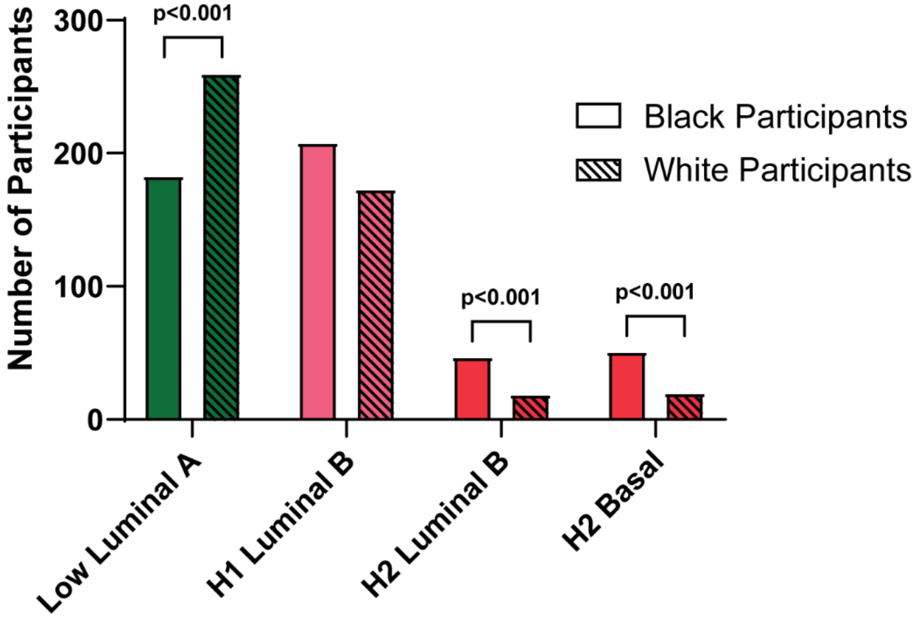

Supplemental Figure 1. Number of participants with MammaPrint and BluePrint tumors by race. Combined MammaPrint (MP) and BluePrint (BP) subtype distribution in Black (N=492) and White (N=475) females, stratified by MP Low Risk/BP Luminal A-Type (dark green), MP High 1/BP Luminal B-Type (pink), MP High 2/BP Luminal B-Type (red), and MP H2/BP Basal-Type (red). Significance between groups were assessed using Pearson's Chi-squared tests. Statistical significance was defined as $p<0.05$. Participants with genomically HER2-Type tumors were removed from analyses due to the small number of participants with available data (N=5). Abbreviations: MP, MammaPrint; BP, BluePrint; H1, MP High 1; H2, MP High 2; N, number of participants.

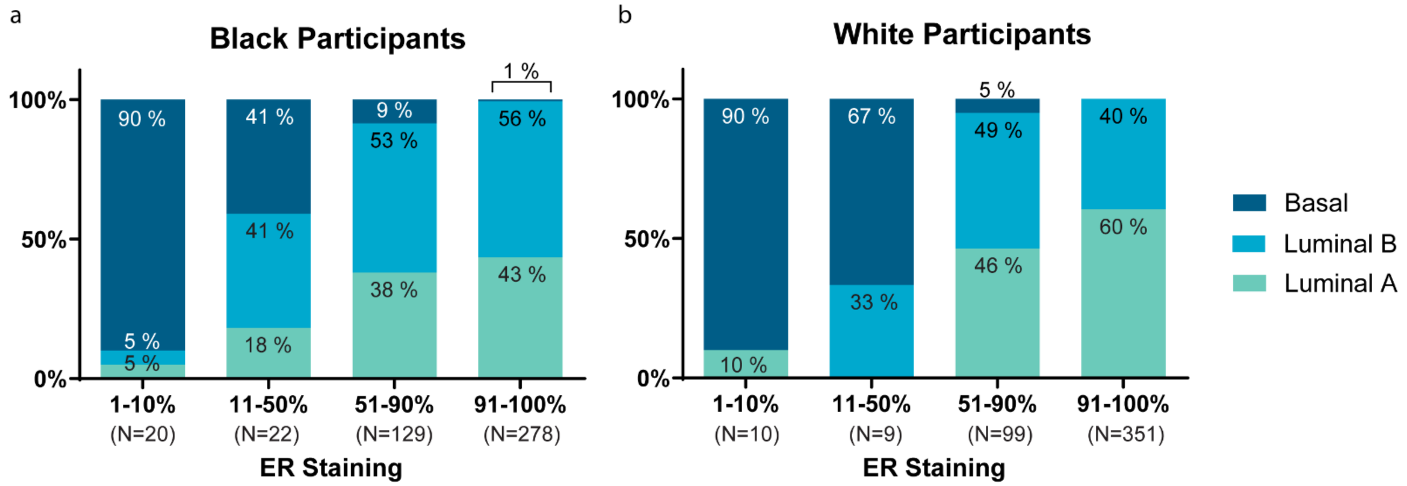

Supplemental Figure 2. Distribution of BluePrint (BP) molecular subtypes by estrogen receptor (ER) staining category and race. Proportional distribution of BP molecular subtypes among (A) Black and (B) White participants stratified by ER immunohistochemistry (IHC) staining categories (1–10%, 11–50%, 51–90%, and 91–100%). Bars represent 100% of participants within each ER category, with segments corresponding to BP Basal, BP Luminal B, and BP Luminal A subtypes. For Black participants, total sample sizes by ER category were: 1–10% (N=20), 11–50% (N=22), 51–90% (N=129), and 91–100% (N=278). For White participants, total sample sizes by ER category were: 1–10% (N=10), 11–50% (N=9), 51–90% (N=99), and 91–100% (N=351). Percentages shown within each bar segment represent the proportion of participants within each ER category and are rounded to the nearest whole number.
